# *Dirofilaria repens* microfilariae from a human node fine-needle aspirate: a case report

**DOI:** 10.1186/s12879-016-1582-3

**Published:** 2016-06-06

**Authors:** Lucia Fontanelli Sulekova, Simona Gabrielli, Maurizio De Angelis, Giovanni L. Milardi, Carlo Magnani, Biancamaria Di Marco, Gloria Taliani, Gabriella Cancrini

**Affiliations:** “Dipartimento di Medicina Clinica (Clinica Malattie Infettive e Tropicali)” “Sapienza” Università di Roma, Rome, Italy; Policlinico Umberto I, Viale del Policlinico, 155, 00161 Rome, Italy; “Dipartimento di Sanità pubblica e Malattie infettive (Unità Operativa Dipartimentale di Diagnostica delle Parassitosi)” “Sapienza” Università di Roma, Piazzale Aldo Moro 5, 00185 Rome, Italy; Ospedale Civile di Legnano, Via Papa Giovanni Paolo II, 20025 Legnano, MI Italy

**Keywords:** *Dirofilaria repens*, Microfilariae, Fine-needle aspirate, Italy, Microscopy, Molecular diagnostics, Immunodiagnostics

## Abstract

**Background:**

Human dirofilariosis is still a little known infection even in endemic areas. Dirofilariosis is zoonotic infection usually abortive in humans; instead, we report a very rare case (the 4th in the world), the first in Italy, in which at least two infective larvae became mature adults that mated and produced active microfilariae even though they did not reach peripheral blood.

**Case presentation:**

A 30-year-old Italian woman presented with a transient oedematous swelling on the left abdominal wall with a creeping eruption followed by the occurrence of a subcutaneous nodular painless mass in the iliac region. One month later, a similar temporary swelling appeared on the contralateral inguinal region associated with intermittent joint discomfort in both knees. The patient had recently travelled abroad, therefore many possible diagnoses were to be ruled out. Routine laboratory investigations revealed eosinophilia. An ultrasound examination of the iliac swelling evidenced a well-defined cyst with a big filamentous formation in continuous movement. A fine-needle aspiration of the lesion was performed for parasitological, cytological and histological exams. The prompt microscopic examination of the aspired material showed the presence of numerous microfilariae that were initially morphologically attributed to *Mansonella ozzardi.* Subsequently, the revision of the Giemsa stained film and molecular analyses of the biological material, allowed to identify *Dirofilaria repens* as etiological agent of infection.

**Conclusions:**

We report of a case in whom microfilariae were detected in fine-needle aspirate of subcutaneous node, without evidence of microfilaraemia, and the infection failed to become fully patent. Therefore we confirm that complete development and fertilization of *D. repens* worms in human hosts may occur, at variance with what is commonly believed, that *Dirofilaria* worms cannot fully develop in humans.

**Electronic supplementary material:**

The online version of this article (doi:10.1186/s12879-016-1582-3) contains supplementary material, which is available to authorized users.

## Background

Dirofilariae are Onchocercidae nematodes that affect domestic and wild carnivores living in tropical and temperate regions of the World, where they are transmitted at the end of a mosquito blood meal, when infective larvae L3 leave the insect and penetrate into the skin. The most important vectors in Italy and in many European countries are the opportunistic feeders *Aedes albopictus* and *Culex pipiens* [1], that may transmit the infection also to humans who are being increasingly found affected by this zoonosis [2]*.* However, the L3s, which in animals develop to adult worms producing microfilariae that circulate in the bloodstream, in humans (not fully suitable hosts) only seldom reach the adult stage, and more rarely the developed adult worms meet, mate and yield microfilariae that, have been exceptionally reported in the blood [3–5]*.* Indeed, the penetration of active larvae is usually followed by a considerable antibody response that aborts most infections. The threadlike 1 mm-15 cm long worm often reaches the final location after long-lasting migrations through the human body and, easily detected in ocular locations, is barely suspected when is blocked in subcutaneous or in asymptomatic more internal tissues.

As for the *Dirofilaria repens* infection, it is usually characterized by the occurrence, 2–12 months after the L3 penetration, of a single subcutaneous nodule, often accompanied by local erythema, pruritus or urticarial manifestations. Usually, it hosts only one immature female, while nodule with one male specimen, much shorter and thinner, is rarely detected. In both cases, infection is devoid of specific characteristics, therefore deeply located nodules are often misidentified as malignant tumours, requiring invasive investigation and surgery before being correctly diagnosed.

We report the case observed in a traveller, firstly attributed to mansonellosis*.* Amended morphological and molecular identification of *Dirofilaria repens* was made on microfilariae detected only in a node aspirate, which is quite an exceptional event.

## Case presentation

**Fig. 1:**
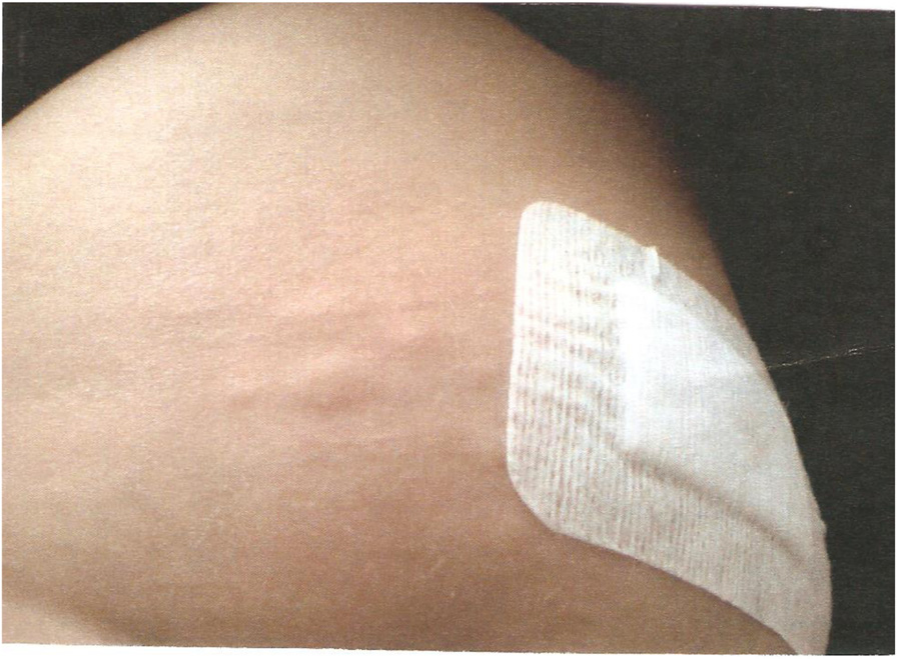
Pruritic dermatitis and swelling with rapid extension to the adjacent areas, observed one hour after the fine-needle aspiration of the subcutaneous node appeared in the inguinal region of the patient

In September 2014, a 30-year-old woman, resident in northern Italy, was referred to Umberto I University Hospital in Rome with a diagnosis of mansonellosis. She reported a month’s stay in India in 2006, in Santo Domingo in 2012 and in Australia in February 2014. In April 2014 a transient oedematous swelling on the left abdominal wall was noticed, with a creeping eruption followed by the occurrence of a subcutaneous nodular painless mass (≈1.5 cm) in the iliac region. Routine laboratory investigations resulted within normal ranges, and ultrasound examination of the node showed nonspecific inflammation. In May, analogue temporary swelling appeared on the contralateral inguinal region, associated with local itching, reddening and intermittent joint discomfort in both knees. Biochemical values were within the normal range, whereas WBC count was 7300/μl with 1100 eosinophils/μl (15.7 %). Lyme disease was suspected due to the presence of erythema migrans skin lesions and arthralgias: however, an enzyme-linked fluorescent assay (ELFA) was performed and resulted negative. A month later, an ultrasound examination of the iliac swelling evidenced a well-defined cyst (0.9 × 0.7 cm) with uniform anechoic content and a big filamentous formation in continuous movement. A fine-needle aspiration of the lesion was performed for parasitological, cytological and histological exams. One hour after this procedure, in the same area, pruritic dermatitis and swelling with rapid extension to the adjacent areas were observed (Fig. 1), which regressed after antihistamines administration. The prompt microscopic wet examination of the aspired material revealed very small mobile elements compatible with microfilariae (Additional file 1). A filariosis was suspected and the patient started doxycycline 100 mg, bid. Cytological examination reported acute inflammation. After Giemsa staining, microfilariae were attributed to *Mansonella ozzardi.* For this reason the patient was treated with ivermectin, 6 mg. A second dose of ivermectin (6 mg) was administered 14 days later. Doxycycline was interrupted after a 14 days course. In July, the same symptomatology occurred about 10 cm aside, eosinophilic count was normal, and ultrasound analysis evidenced static remains of a worm. The patient was then referred to Tropical Medicine Unit of the Umberto 1° University Hospital in Rome.

On first examination, the patient was asymptomatic and her physical examination was negative. Nevertheless, the following investigations were performed: i) revision of the Giemsa stained film; ii) molecular analyses of the biological material previously sent for histological analysis; iii) searching for microfilariae in peripheral blood; iv) serological test to detect reactivity to filarial antigens.

Slides were revised by microscopy; microfilariae were measured, and identified based on their morphological features. DNA was extracted from the paraffin block according to a previously reported method [6]*,* and a *cox1* (about 650-bp) gene fragment was PCR-amplified by using filarioid-generic primers as previously described [7]*.* The amplicon was purified and sequenced; sequences were aligned using ClustalW program and compared with those available in GenBank (http://blast.ncbi.nlm.nih.gov/Blast.cgi). Finally, a blood sample was obtained on which the search for microfilariae by microscopy (on thick smears Giemsa stained after Knott’s concentration) and by PCR and finally testing for antibodies to antigens of Onchocercidae filariae by means of an ELISA test (*Acanthocheilonema vitae,* Bordier Affinity Products, Crissier, CE) were performed. The sensitivity and specificity of the ELISA test are respectively 95 and 98 %. It is important to note that this test does not differentiate between different filarial infections.

**Fig. 2:**
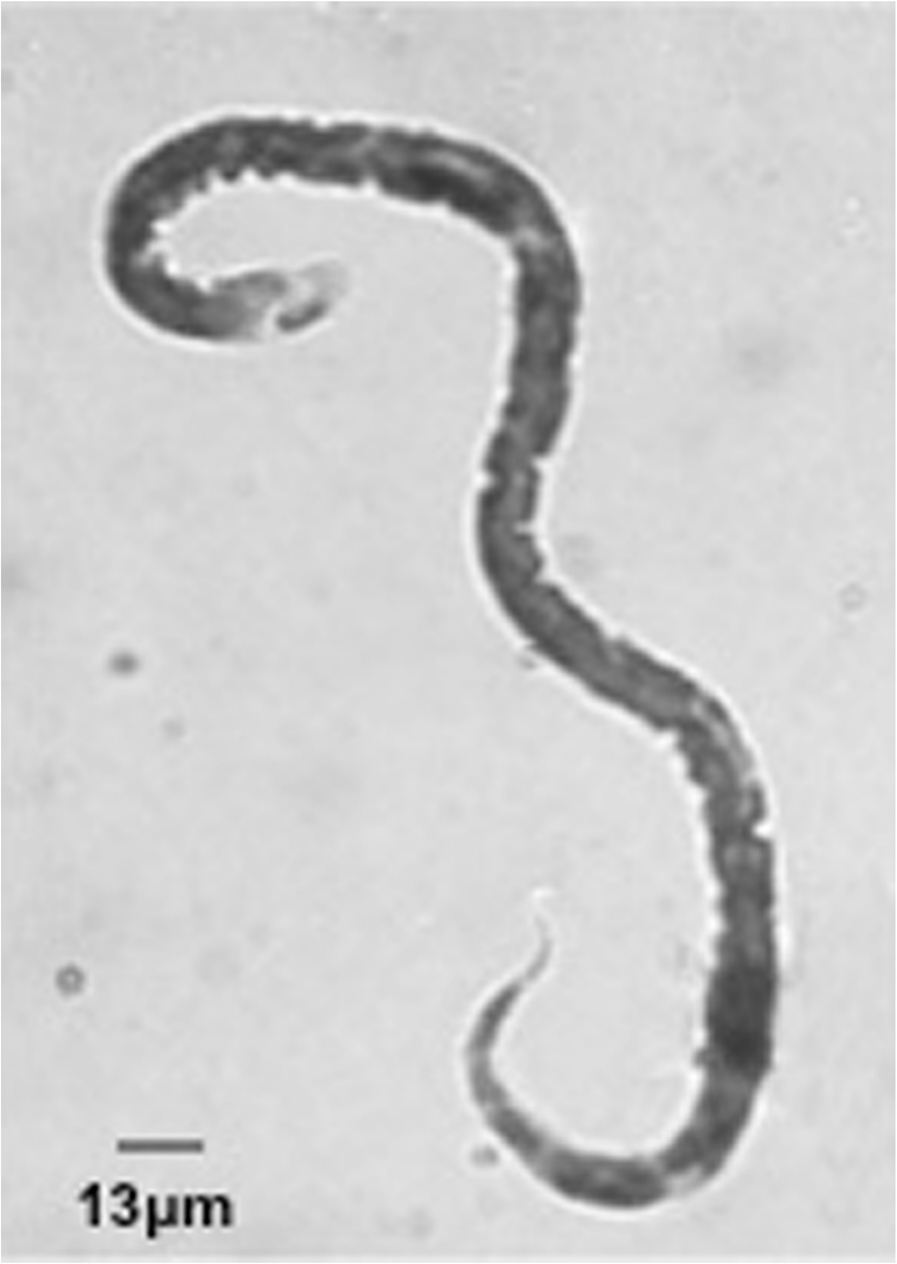
Giemsa stained microfilaria identified before as *Mansonella ozzardi* and then as *Dirofilaria repens.* Length, wideness and caudal end curved in the form of an umbrella handle are the diagnostic features

The revision of the slide previously considered positive for *M. ozzardi* showed many microfilariae sized 312-350 × 7.5 μm, without sheath, with an obtuse cervical end and a sharp threadlike caudal end curved in the form of an umbrella handle (Fig. 2). These features, incompatible with morphology of *M. ozzardi,* are fully compatible with that of the zoonotic *D. repens,* as confirmed by molecular analyses: sequences obtained (accession number KT899073) evidenced 100 % identity with the *cox1* sequence of *D. repens* (accession number DQ358814.1). Extensive examinations on the peripheral blood, including molecular testing, failed to detect circulating microfilariae. Finally, serology for Onchocercidae filariae confirmed the reactivity to filarial antigens.

An ultrasound examination of the node one month after the biopsy and treatment with doxycycline and ivermectin did not show any images compatible with living parasites and physical examination did not point out any nodule to remove, therefore we did not pursue any surgical treatment, and we did not prescribe any antihelmintic drugs. On May 2015, 8 months after *D. repens* was diagnosed, WBC count was 9200/μl with eosinophils 160/μl (1,75 %). Nowadays, more than a year after *D. repens* diagnosis, the patient occasionally suffers from transient urticaria-like symptoms, mild inguinal pain and knees arthralgia, symptoms that are likely due to *D. repens*. She is still on follow-up to evaluate if new signs of parasitic infection, such as eosinophilia or evidence of new nodule develope.

## Discussion

About half of the human dirofilarioses identified in Europe are reported from Italy, and the case presented here occurred in an endemic area where many other cases have been identified. Nevertheless, there is a widespread lack of awareness for this zoonosis, and reliable diagnostic tests are applied only in few specific structures. Indeed, since the patient had travelled abroad, anthroponotic mansonellosis was a possible diagnostic option. The clinical progress of the infection included an initial migration of the parasite and the following development of a subcutaneous nodular painless mass. Early ultrasound examination of the node detected filamentous formation in continuous movement, a rare finding [8–10] that suggested the presence of an adult filaria and advised further investigations until the diagnosis of *D. repens* was made. However, when the patient was referred to the Policlinico University Hospital she was asymptomatic and physical examination was negative.

To the best of our knowledge, no data are available on the effects of ivermectin and doxycycline therapy against *D. repens.* Studies carried out on some anthroponotic filariae and on *Dirofilaria immitis* obtained discordant results. However, it seems that only doxycycline may have a possible adulticidal effect [2, 11]*,* due to its action on the bacterial endosymbiont *Wolbachia*, present in most filarial species (*D. repens* included). It is conceivable that doxycycline had an adulticidal effect and a role in the absence of microfilariae in the bloodstream, but it should be taken in mind that the patient received only a brief course of treatment (14 days), and peripheral blood was tested for microfilariae 3 months after therapy withdrawal.

Should a new nodule appears, we will consider the possibility of removing the nodule. Should surgical removal be difficult to perform, we will prescribe a 6-week treatment with doxycycline, given its possible adulticidal effect.

The described case is remarkable for at least three reasons: firstly, from a biological point of view, since in humans rarely more than one adult develops, and only exceptionally mature worms meet, mate and generate active microfilariae (unusual immunotolerance of the unsuitable host). Secondly, the new-borne larvae remained inside the nodule without reaching the peripheral blood, as previously reported in only three cases [12–14], which makes the infection not fully patent*.* Finally, this is the first case of human dirofilariosis diagnosed in Italy based on microfilariae (by microscopy and molecular assays), therefore laboratories must carefully consider this diagnostic possibility when dealing with microfilariae. Indeed, as hypothesized by GIS models [15]*,* this zoonosis is spreading. Cases are increasingly reported, and are found in areas where had never occurred previously, due to the global warming and drivers (i.e. increasing number of pets, parallel practice of animal abandoning, travelling in endemic areas with pets, and expanding urbanisation) that favour the parasite spreading and encourage the migration of the mosquito vectors from too warm areas to cooler habitats.

## Conclusions

Even if Italy is the Country from which there have been reported about half of the cases of human dirofilariosis identified in Europe, there is a widespread lack of awareness of this parasitic disease, and reliable diagnostic tests are applied in few specialistic laboratories. Dirofilariosis should be included in the differential diagnosis in patients presenting subcutaneous nodules and clinicians must carefully consider this diagnostic possibility when dealing with microfilariae.

## Abbreviations

GIS, geographic information system; PCR, Polymerase chain reaction; WBC, white blood cells; bid: bis in die

## Additional file

Additional file 1: Alive elements compatible with microfilariae, fine-needle aspirated from a subcutaneous node and detected at the microscopic examination of the material. (mov 914 kb)
